# Autopsy in adults with congenital heart disease (ACHD)

**DOI:** 10.1007/s00428-020-02779-8

**Published:** 2020-04-07

**Authors:** Annalisa Angelini, Cira di Gioia, Helen Doran, Marny Fedrigo, Rosa Henriques de Gouveia, Siew Yen Ho, Ornella Leone, Mary N Sheppard, Gaetano Thiene, Konstantinos Dimopoulos, Barbara Mulder, Massimo Padalino, Allard C van der Wal, On behalf of Association for European Cardiovascular Pathology (AECVP)

**Affiliations:** 1 Cardiovascular Pathology, Department of Cardiac, Thoracic, Vascular Sciences and Public Health, University of Padua, Padua, Italy; 2 Department of Radiological, Oncological and Pathological Sciences, Sapienza, University of Rome, Rome, Italy; 3 Department of Pathology, Manchester Foundation Trust Wythenshawe Hospital, Manchester, UK; 4 Department of Pathology, Hospital de Santa Cruz (CHLO), Lisbon & Forensic Pathology, INMLCF & FMUC, Coimbra, Portugal; 5 Royal Brompton Hospital and National Heart and Lung Institute, Imperial College London, London, UK; 6 Department of Pathology, Sant’Orsola-Malpighi University Hospital, Bologna, Italy; 7 Department of Cardiovascular Pathology, St Georges Medical School, London, UK; 8 Adult Congenital Heart Centre and Centre for Pulmonary Hypertension, Royal Brompton Hospital and National Heart and Lung Institute, Imperial College London, London, UK; 9 Amsterdam University Medical Centers, University of Amsterdam, Amsterdam, Netherlands

**Keywords:** Autopsy, Cardiovascular pathology, Congenital heart diseases, Adult congenital heart diseases, Protocol

## Abstract

The adult congenital heart diseases (ACHD) population is exceeding the pediatric congenital heart diseases (CHD) population and is progressively expanding each year, representing more than 90% of patients with CHD. Of these, about 75% have undergone surgical and/or percutaneous intervention for palliation or correction. Autopsy can be a very challenging procedure in ACHD patients. The approach and protocol to be used may vary depending on whether the pathologists are facing native disease without surgical or percutaneous interventions, but with various degrees of cardiac remodeling, or previously palliated or corrected CHD. Moreover, interventions for the same condition have evolved over the last decades, as has perioperative myocardial preservations and postoperative care, with different long-term sequelae depending on the era in which patients were operated on. Careful clinicopathological correlation is, thus, required to assist the pathologist in performing the autopsy and reaching a diagnosis regarding the cause of death. Due to the heterogeneity of the structural abnormalities, and the wide variety of surgical and interventional procedures, there are no standard methods for dissecting the heart at autopsy. In this paper, we describe the most common types of CHDs that a pathologist could encounter at autopsy, including the various types of surgical and percutaneous procedures and major pathological manifestations. We also propose a practical systematic approach to the autopsy of ACHD patients.

**Electronic supplementary material:**

The online version of this article (10.1007/s00428-020-02779-8) contains supplementary material, which is available to authorized users.

## Introduction

Over the last 40 years, major advances have been made in the clinical diagnosis and treatment of congenital heart diseases (CHD) [1]. These developments have resulted in an increasing population of patients who survive into adulthood – the adults with congenital heart disease (ACHD). ACHD includes a large variety of congenital disorders [2]. Nowadays, the ACHD population is exceeding the pediatric CHD population and is expanding by an estimated 5% per year. More than 90% of patients with CHD are adults, and of these, about 75% have undergone palliative intervention or surgical correction [3–7].

Approximately 50% of ACHD patients face complications during their lifetime, depending on the type of defect and previous interventions, most commonly arrhythmias, ventricular dysfunction and heart failure, endocarditis, need for reoperation (e.g., due to valve or conduit dysfunction), pulmonary hypertension, and premature or sudden cardiac death (SCD) [8]. Early diagnosis and treatment has altered the natural history of CHD and has led to new and, at times, complex types and patterns of cardiovascular and lung pathology that increasingly require the attention of expert pathologists.

Autopsy can be a very challenging procedure in ACHD patients. The approach and protocol may vary depending on whether we are faced with cases of native CHD who have reached adulthood without surgical or percutaneous interventions (but with various degrees of cardiac remodeling) or cases of previously palliated or surgically/percutaneously corrected conditions.

Careful clinicopathological correlation is, thus, required to assist the pathologist in performing the autopsy and reaching a diagnosis regarding the cause of death. It is essential that the clinical history, including detailed surgical reports, is available to the pathologists at the time of the autopsy.

Clinicians and/or family members who request an autopsy may wish to obtain information on one or more of the following questions:

- Was the clinical diagnosis correct?
- Were there any associated abnormalities/comorbidities not diagnosed prior to an intervention that could have impacted on its outcome?
- Was a surgical or interventional procedure (correction or palliation) performed correctly?
- Was the timing of intervention appropriate?
- Were there any unrecognized complications contributing to the death of the patient?

For this purpose, a pathologist who performs such an autopsy must have good knowledge of the following:

- *Morphology of CHDs*: Sequential segmental analysis and knowledge of flow patterns through the morphologically abnormal heart
- *Past and contemporary surgical procedures:* For example, palliative shunts, vascular grafts, and patches; knowledge of flow patterns in the operated heart; potential complications
- *Percutaneous interventional procedures*: *For example, stents, occluder devices, percutaneous valve prostheses*
- *Postoperative pathology*: Residual lesions short- and long-term complications (e.g., valve degeneration, thrombosis, embolic events, endocarditis)
- *Patterns of heart remodeling*: *Hypertrophy, dilatation and fibrosis, associated with arrhythmia, and heart failure*
- *Related pathology in other organs*: Pulmonary edema, pulmonary vascular disease, protein loosing enteropathy, liver cirrhosis, kidney injury, cerebral abscess
- *Syndromic CHD and associated extracardiac malformations*

Due to the heterogeneity of the structural abnormalities mentioned above, and the wide variety of surgical and interventional procedures, there are no standard methods for dissecting the heart at autopsy. In this paper, we describe the most common types of CHDs that a pathologist could encounter at autopsy, including the various types of surgical or interventional procedures and major pathological manifestations. We also propose a practical systematic approach to the autopsy of ACHD patients.

## The most common congenital heart defects encountered in adults

### Atrial septal defects (ASDs)

Description: ASD is a direct communication between the atria allowing shunting of blood.

**Fig. 1:**
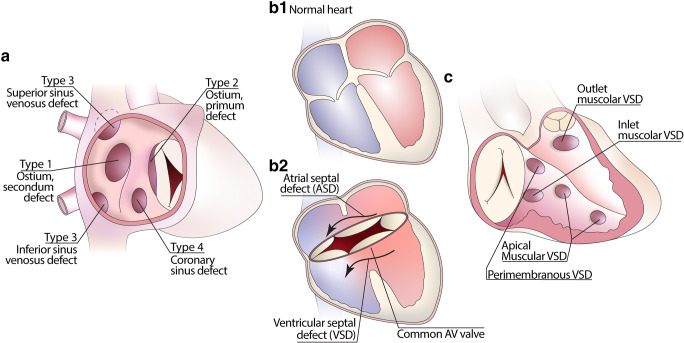
Drawings of the most common types of atrial (**a**), atrioventricular (**b**) and ventricular septal defects (**c**), and their locations inside the cavities

There are four types of ASD, depending on the location of the defect (Fig. 1a):

- *Type 1: Ostium secundum type ASD*. This is the most common type of ASD. The defect is located within the fossa ovalis (FO), which is about 1–2 cm in diameter, and often remains undiagnosed until later in life.
- *Type 2: Ostium primum defects*. These are located inferiorly close to the atrioventricular valve and are separated from the FO by its inferior muscular rim. This type of ASD is typically part of the spectrum of atrioventricular septal defects and is associated with abnormal atrioventricular valves (see also atrioventricular septal defect section)
- *Type 3: Sinus venosus ASD*. This can be a superior or an inferior sinus venosus type of ASD, being related to the orifice of either the superior or the inferior vena cava (SCV or IVC), respectively. The superior variant is commonly associated with partially anomalous pulmonary venous connection [9]. The inferior defect is positioned close to the orifice of the ICV and can be associated with an anomalous right inferior pulmonary vein.
- *Type 4: Coronary sinus defect*. It is very rare and located closely to the ostium of the coronary sinus (CS). It is due to deficiency of the walls of the coronary sinus allowing shunting with the left atrium (LA) [10].

Most ASD cases are sporadic and very few run in families in an autosomal dominant pattern [11]. Ostium primum defects are common in individuals with Down syndrome or Ellis-Van Creveld syndrome. One third of ASD occur in association with other cardiac malformations [12].

Symptoms related to ASDs depend on the size of the defect and associated lesions/comorbidity. Many patients remain asymptomatic for many years and may remain undiagnosed throughout their lives, while others present in childhood. Many ASDs are nowadays diagnosed after the age of 40 years. Symptoms include arrhythmia, paradoxical embolism, right ventricular failure, and, more rarely, cerebral abscess and pulmonary hypertension.

ASD causing significant left-to-right shunting or other complications should be repaired, unless pulmonary vascular disease has developed. Most cases of secundum ASD are nowadays closed percutaneously, using an occluder device.

Failure to align the device can result in device embolization and is likely to occur in very large defects (>/ = 35 mm), with absent or deficient posterior or inferior rim. Moreover, percutaneous closure may be challenging in cases with a multi- fenestrated and/or aneurysmal atrial septum or proximity of the FO to the venous orifices [13, 14] (Table S2 supplement). When a transcatheter approach is not feasible or a primum or sinus venosus ASD is present, open-heart surgery is required to close the defect with a graft or prosthetic patch [15, 16] (Table 1).

**Table 1:** Major congenital cardiac defects and autopsy considerations after surgical procedures

Type of defects	Considerations after surgical procedures
ASD	Residual defect after patch or device closure Size of the right and left atria Size of the right and left ventricular chambers Pulmonary vascular disease
AVSD	Relative size of the ventricles: unbalanced type, with one dominant and one hypoplastic ventricle Left and right atrioventricular valve incompetence Left and right atrioventricular valve dysplasia Subaortic or subpulmonary obstruction due to left atrioventricular valve replacement or ventricular patch Residual VSD or ASD Pulmonary vascular disease Complete heart block Sudden death Endocarditis
VSD	Residual defect after patch or device closure AV node/conduction tissue damage/dysfunction Remodeling of the right or left ventricle Aortic or tricuspid valve incompetence Endocarditis
Truncus arteriosus	RV-PA conduit stenosis, incompetence or infection Truncal valve regurgitation, stenosis or infection Prosthetic valve dysfunction or endocarditis Coronary arteries anomalies leading to myocardial damage Residual VSD Truncal root dilatation Pulmonary artery stenosis Pulmonary vascular disease
Tetralogy of Fallot	Pulmonary valve regurgitation Residual VSD Residual pulmonary/ right ventricular outflow tract stenosis Ventricular or atrial tachyarrhythmias Endocarditis RV-PA conduit dysfunction Aortic root dilatation (± aortic valve regurgitation) Right and/or left ventricular dysfunction Congestive heart failure
Congenital aortic valve disease	Aortic valve stenosis or incompetence LV hypertrophy, dilatation, and/or dysfunction Aortic dilatation Ross procedure: pulmonary valve prosthesis, coronary artery re-implantation, dilatation of the neo-aorta Bentall procedure: coronary artery re-implantation Conduction abnormalities Endocarditis
Aortic coarctation	Re-coarctation (most common with end-to-end or percutaneous repair) Associated defects: bicuspid aortic valve, subaortic stenosis, mitral valve disease, ascending aortic dilatation. (Pseudo) aneurysms at the site of previous repair Infective endocarditis or arteritis Cerebral berry aneurysm rupture Aortic dissection Premature coronary artery disease Left ventricular hypertrophy/remodeling
(D-)TGA	*After Mustard or Senning procedure*: systemic ventricular morphology, scarring, tricuspid valve abnormalities, pathway obstruction, baffle leaks, pulmonary stenosis, pulmonary vascular disease, subpulmonary LV size and function *After arterial switch procedure*: Coronary artery abnormalities/distortion, neo-aortic root dilatation, neo-aortic valve regurgitation, right ventricular outflow tract obstruction, ventricular remodeling
Pulmonary valve disease	Pulmonary/ prosthetic valve stenosis or incompetence Right ventricular remodeling Right ventricular scarring Aneurysmal dilatation of the pulmonary artery Endocarditis

#### Autopsy recommendations

Autopsy should identify the location, type, and size of an unrepaired ASD, as well as associated lesions. Moreover, dilatation of the RA and LA, tricuspid valve annular dilatation, and dilatation (and at times hypertrophy) of the right ventricle (RV) are typically encountered. Pulmonary artery dilatation is common, while atheromatous changes in the pulmonary arteries and significant RV hypertrophy suggest pulmonary hypertension. Histology of the lungs is essential to detect vascular changes.

In repaired ASDs, the position and size of surgical patches and closure devices, as well as residual communications, should be described. Displacement or embolization of a device and dehiscence of a surgical patch should be excluded. After timely successful repair, the heart is expected to remodel toward normal. Both patches and devices become endothelialized 6–12 months after intervention and may be difficult to identify. Late complications include thrombosis of the device and embolic phenomena (stroke or coronary artery and systemic embolization), complete heart block, and infective endocarditis. Moreover, rare cases of device erosion toward the aorta have been described [17] (Table 1, Table S2 supplement).

#### Atrioventricular septal defects (AVSD)

Description: AVSDs (synonyms: atrioventricular canal defects or endocardial cushion defects) are characterized by a common atrioventricular junction and a common atrioventricular valve resulting in communication between the atria and ventricles and all four cardiac chambers, depending on the anatomical severity (Fig. 1b). Down’s syndrome is commonly associated with AVSDs, present in one third to one half of all patients.

#### There are several types of AVSDs

The *ostium primum defect (or incomplete AVSD)* (see also ASD section) has a common atrioventricular junction. The bridging leaflets of the atrioventricular valve are joined together and are adherent to the crest of the ventricular septum forming two separate valve orifices that are often regurgitant. As a result, shunting across the defect occurs only at atrial level [18]. Surgery abolishes the shunt and associated overload to the RV while repairing or, more rarely, replacing the atrioventricular valves [19].

The *complete form of AVSD* is characterized by having a communication allowing both interatrial and interventricular shunting, a common atrioventricular valve orifice and a five-leaflet valve, most often dysplastic, but with the bridging leaflets not adherent to the atrial septum or the crest of the ventricular septum. Surgical correction with closure of interatrial and interventricular components, along with repair and reconstruction of atrioventricular valves, is done. After repair of an AVSD, a re-intervention may be needed for left-sided valve insufficiency or stenosis, often years after repair, with rate of re-intervention of about 10% [20].

Complete AVSDs can be classified into balanced and unbalanced according to the relative dimensions of the ventricles. In unbalanced AVSDs, one of ventricles is too small/hypoplastic and may be deemed unsuitable for a biventricular repair. These cases require a staged single-ventricle palliation with a Fontan or Fontan-like procedure [20] (Table 1).

#### Autopsy recommendations

Unoperated cases are almost never seen in the adult population and are usually partial AVSDs (primum ASDs). Adults with unrepaired complete AVSDs typically present with severe pulmonary vascular diseases (Eisenmenger syndrome).

In repaired cases, patches and sutures are usually re-endothelialized and calcified for months or years following surgery. The presence of residual VSD and ASD, left-sided valve pathology (stenosis or regurgitation, surgery, endocarditis), subaortic or pulmonary stenosis, and myocardial remodeling of atria and ventricles with histologic examination of fibrosis (risk of arrhythmia) all needs to be evaluated at autopsy. Finally, pulmonary vascular disease and endocarditis should be excluded. In palliated cases, the presence of banding of the pulmonary trunk or a Fontan-type circulation (see later) should be described (Table 1).

### Ventricular septal defects (VSDs)

Description: A group of common congenital heart defects characterized by holes in the ventricular septum.

There are several types of VSD (Fig. 1c): A *perimembranous* has the membranous septum incorporated into its postero-inferior border. *Muscular VSDs* are completely surrounded by muscle and can be located anywhere in the muscular ventricular septum. *Subarterial VSDs* (synonyms: supracristal, doubly committed or juxta arterial defects) are immediately adjacent to both the aortic and pulmonary valves and may have perimembranous or muscular border.

Patients with certain genetic abnormalities, e.g., Down syndrome, have a high incidence of associated VSDs: VSD is an integral part of tetralogy of Fallot, and VSDs are present in most patients with univentricular circulation or transposition of the great arteries.

VSDs with a maximal size of 2 mm or less are likely to close prenatally, while others may close within the first 10 years of life [21]. Residual scarring in the septum or scarring with aneurysmal dilatation of the membranous septum with redundant tricuspid valve tissue may be seen at autopsy at the site of spontaneously closed defect.

Persistent VSDs are often restrictive but may be larger. Large or multiple VSDs can lead to pulmonary hypertension and right ventricular hypertrophy, eventually with the development of Eisenmenger syndrome characterized by shunt reversal and cyanosis. Patients with a VSD and significant, irreversible pulmonary vascular disease are deemed inoperable.

For this reason, timely surgical closure is recommended for all large perimembranous VSDs, supracristal VSDs, and VSDs with aortic valve prolapse [22]. Muscular VSDs may be closed by percutaneous techniques. A large number of devices have been used for VSD occlusion, the Amplatzer VSD occlude device being the most popular (Table 1).

#### Autopsy recommendations

Surgical patches can be calcified and fibrosed, or largely incorporated into the ventricular septum many years after surgery, and can be difficult to identify. Postoperative complications that should be searched for are the following: dehiscence of the patch, heart block in perimembranous septal defects (due to surgical damage of left bundle branch), infective endocarditis (operated and non-operated cases), and patch-related thrombus or embolization. In case of supracristal defects, accompanying aortic regurgitation is common, produced by prolapse of the anterior aortic leaflet. Residual VSDs should be identified and described (e.g., suture dehiscence). Muscular VSDs may be more difficult to identify. Remodeling of the LV and RV should be described, and lung histology is required to identify and grade pulmonary vascular disease. The presence of pacemakers for permanent pacing may indicate manifestations of complete heart block during life (Table 1, Table S2 supplement).

### Truncus arteriosus or common arterial trunk

Description: The *common arterial trunk (CAT*), also known as *truncus arteriosus*, is a rare type of CHD (1–3% of cases) and therefore mentioned only briefly here.

The anomaly is characterized by a single (common) arterial trunk overriding a large VSD, supplying the coronary, systemic, and pulmonary arterial system. The latter arises from the common trunk either as separate right and left pulmonary arteries or as a single pulmonary trunk that subsequently bifurcates. This common trunk is guarded by a truncal valve, which is always in continuity with the mitral valve. In most cases, there is usual atrial arrangement (situs solitus) and concordant atrioventricular connections. In majority of cases, the truncal valve has three leaflets and is grossly dysplastic and incompetent, but it may also be stenotic. Approximately 30% of truncus arteriosus cases are associated with a genetic syndrome, frequently DiGeorge syndrome with 22q11deletion. The most significant variation in the aortic pathways associated with CAT is severe coarctation or complete interruption of the aortic arch, with the proximal aorta arising directly from the common trunk and the distal aorta fed through a patent arterial duct.

Without surgical repair, most truncus arteriosus patients die in early life, most frequently due to severe pulmonary arterial hypertension (PAH) or from heart failure and associated low flow conditions such as myocardial ischemia and necrotizing enterocolitis.

Surgical repair is indicated in the neonatal period, or by 2–3 months of age due to the rapid progression of pulmonary vascular disease. Repaired cases achieve an 80% long-term survival [23]. The aim of surgical repair is to restore a normal physiologic circulation. The pulmonary arterial trunk (or branches) is detached from the truncus and attached to the right ventricle (RV) via a homograft (a valve conduit). The VSD is closed with a patch. The truncal valve becomes the neo-aortic valve, and, if necessary, it is repaired or replaced by an aortic homograft with coronary artery re-implantation (Table 1).

#### Autopsy recommendations

In surgically repaired CAT adult patients, the pathologist has to look for a possible residual VSD in relation to the patch, truncal valve regurgitation or stenosis due to dysplastic leaflets, or prosthetic valve dysfunction, aortic (truncal) root dilatation, coronary artery abnormalities, stenosis or incompetence of the valved conduit right ventricular outflow or pulmonary artery stenosis, and, importantly, endocarditis. Histological examination of the myocardium and lungs is required to evaluate early and late scaring and the presence of pulmonary vascular disease (Table 1, Table S2 supplement).

### Pulmonary stenosis

Description: Pulmonary stenosis is a common type of CHD and is frequently isolated but can also be associated with simple or complex congenital cardiac defects.

The stenosis can be *valvular*, localized at the site of pulmonary valve or its annulus, or, more rarely, in the *subvalvular* (muscular) infundibulum of the RV, or *supravalvular* in the pulmonary trunk or its branches. In *pulmonary valvular stenosis****,*** the valve is typically a dome-shaped diaphragm with a central perforation without recognizable leaflets. *Isolated infundibular stenosis* is frequently associated with a VSD. Restrictive VSDs can be associated with hypertrophic muscular bundles within the RV causing mid-ventricular obstruction (double-chambered RV).

*Supravalvular stenosis* is a circumferential fibrous ring that obstructs the pulmonary trunk, the main pulmonary branches, or even the distal branches with multiple stenoses typical of Williams syndrome.

The stenosis can be relieved by percutaneous dilatation with balloon (angioplasty) or by surgical maneuvers such as pulmonary commissurotomy, complete or partial removal of the malformed leaflets with or without enlargement of the annulus with a transannular patch. The presence of infundibular obstruction can be addressed through the pulmonary valve annulus if not too hypoplastic, or through the tricuspid valve ventriculotomy, which is rarely required nowadays for the resection of the hypertrophic muscle bands, as it is associated with long-term complication (RV dysfunction, ventricular tachycardia). In supravalvular stenosis, it is necessary to enlarge the pulmonary trunk with autologous or heterologous pericardium or with Gore-Tex (Table 1).

#### Autopsy recommendations

In adult patients with unoperated pulmonary stenosis, the pathologist has to evaluate the level at which obstruction occurs. Secondary changes due to the pulmonary valve anomaly are RV hypertrophy, tricuspid valve insufficiency, and RA dilatation. Poststenotic dilatation affects the pulmonary trunk or its left branch. Surgically treated cases should be examined for pulmonary valve incompetence, RV dilatation, and ventricular scarring. After percutaneous or surgical valve implantation, evaluate the position of the valve, residual stenosis, and valve complications such as paravalvular leak, endocarditis, and calcification of the leaflets (Table 1, Table S2 supplement).

### Tetralogy of Fallot

Description: The defining anatomical abnormalities of the tetralogy, as described by Étienne Fallot in 1888, are VSD with overriding aorta, pulmonary stenosis, and right ventricular hypertrophy (RVH).

TOF is the most common cyanotic CHD accounting for up to 9% of CHD [24]. It can be associated with trisomy 21, trisomy 18, trisomy 13, and DiGeorge syndrome, but in most patients, no genetic association can be found. Congenital abnormalities associated with ToF include bicuspid pulmonary valve, left pulmonary artery stenosis, coronary artery anomalies, right-sided aortic arch, anomalous pulmonary venous return, and skeletal abnormalities of the vertebrae and ribs [25–27].

The severity of right ventricular outflow tract obstruction is variable, but when severe, there is right-to-left shunting through the VSD and systemic cyanosis. Most adult cases encountered nowadays in developed countries will have had reparative surgery early in life, with takedown of any previous palliative shunt (Table 1).

**Fig. 2:**
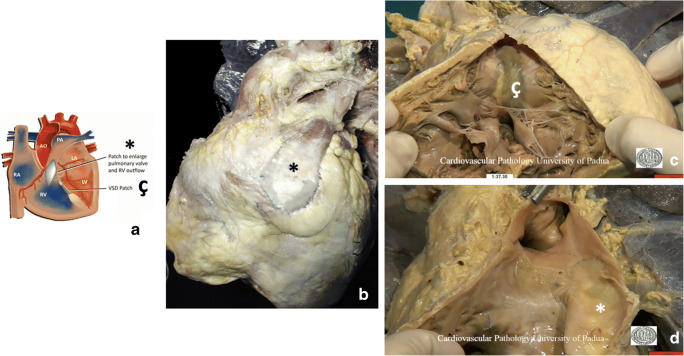
Diagram of ToF after surgical repair, with a patch inserted to enlarge the pulmonary valve and the RV outflow tract (*) and closure of the interventricular defect with a patch (ç) **(a)**. Operated TOF at adult age; the patient died of heart failure **(b, c, d)**. Anterior view of the heart with transannular patch (*) and dilatation of the right ventricle with enlargement of the right heart silhouette **(b)**; internal view of the RV outlet showing the site of the ventricular septal defect closed by an endothelialized patch (ç) and dilatation of the RV **(c)**; close view of the calcified RV outflow patch (*) **(d)**

Definitive repair consists of patch closure of the VSD, with the overriding aorta supplied only by the LV and relief of pulmonary and subpulmonary stenosis by infundibular remodeling and/or a transannular patch. Surgical closure of the VSD may be done through a right ventriculotomy (more traditional approach) or with a transatrial approach through the tricuspid valve (more recent technique) (Fig. 2). Recently, a surgical technique with pulmonary valve reconstruction has been proposed by several authors to avoid or minimize the long-term pulmonary valve regurgitation that is inevitable with the traditional techniques [28–30] (Table 1).

Nowadays, the life expectancy of patients with repaired ToF is felt to be similar to the general population [31], even though there persists a small risk of sudden cardiac death at all ages [32], and pulmonary regurgitation is common which frequently requires further intervention.

Early complications after repair include residual VSD, right ventricular outflow tract obstruction or pulmonary stenosis, and ventricular tachyarrhythmias (Table 1). Later complications include pulmonary regurgitation, progressive dilatation of the aorta with secondary aortic valve incompetence, progressive right ventricular dilatation, or aneurysm, following extensive ventriculotomy, infundibular remodeling, or transannular patching, and tricuspid valve incompetence. Patients with pulmonary atresia, at the extreme end of the spectrum of ToF, require an RV to PA conduits at repair, which typically becomes stenotic or regurgitant over time; a synthetic conduits may develop neointimal hyperplasia, while homograft conduits and valves tend to calcify.

**Fig. 3:**
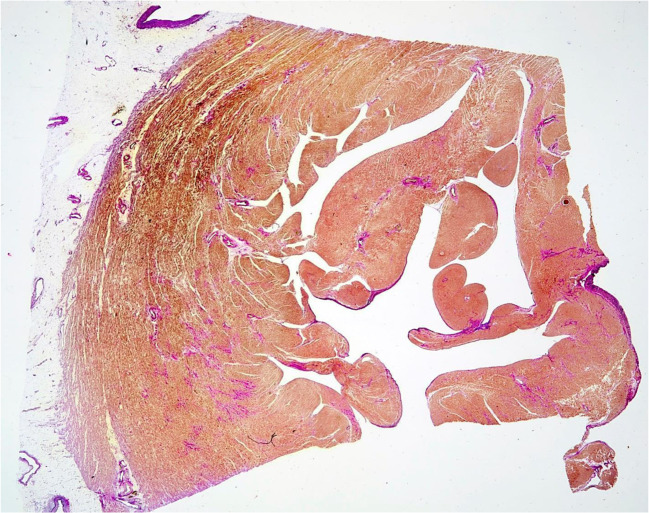
Histology of RV wall of an adult patient with operated ToF. Transmural section showing hypertrophic myocardium and fibrosis of interstitial reactive and replacement type. Elastic van Gieson stain

Previous palliative shunts, right ventricular hypertrophy with fibrosis (Fig. 3), a prolonged QRS duration, ventricular dysfunction, and atrial arrhythmias have been shown to be predictors of death and sustained VT [33]. Patients felt to be at significant risk of sudden death may be offered an ICD as a primary prevention [34].

**Fig. 4:**
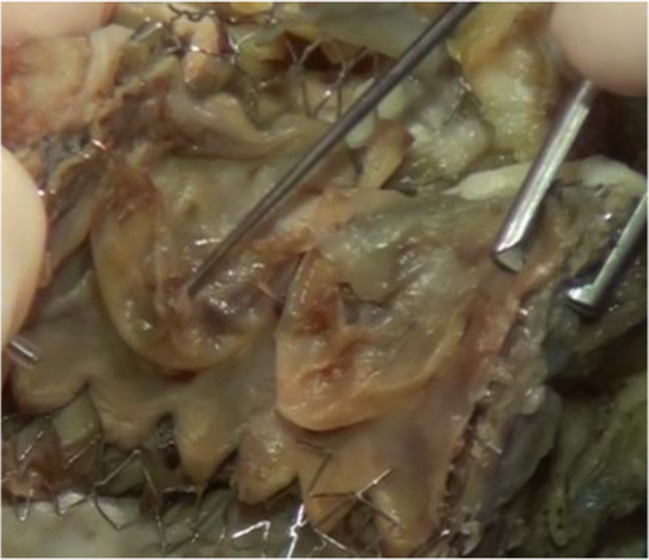
Hybrid procedures in a case of ToF with previous surgery and stenting of the pulmonary valve with a percutaneous intervention, using a *Melody* valve

During adult life, reoperation for pulmonary valve regurgitation is often required in the presence of severe RV dilatation, RV dysfunction, and associated symptoms or progressive tricuspid regurgitation, with very low surgical risk [35]. Several types of bioprosthetic valves, homografts, and, more recently, percutaneous pulmonary valves are used in these patients, depending on availability and anatomical features [36] (Fig. 4) (Table 1, Tables S2 and S3 supplement).

#### Autopsy recommendations

On external examination, there are typically the scars of previous operations, sternotomy or thoracotomy. In patients dying of heart failure, there may be systemic signs of right or biventricular failure, while internally, there may be pleural effusions, pulmonary edema, ascites, and hepatic congestion. Fibrosis and calcification of operation sites, patches, and conduits may be present, with possible stenosis or infection of conduits. Pulmonary and tricuspid valve competence should be assessed, and ventricular size should be measured. Right ventricular hypertrophy and fibrosis may be the substrate for fatal arrhythmia. Endocarditis may be an early or late complication of the original surgery (Fig. 2) or later valve replacements and is often associated with septic emboli in the lung. Percutaneous pulmonary valve implants appear to be prone to infective endocarditis, which is often obstructive. Older patients may have developed superimposed atheroma of native coronary vessels (Table 1, Tables S2 and S3 supplement).

### Left ventricular outflow tract obstruction (LVOTO)

Description: LVOTO in adults includes a series of stenotic lesions which can be single or at multiple sites along the tract. Obstruction can be subvalvular, valvular, and/or supravalvular.

Valvular AS is the most frequent form, accounting for 75% of LVOTO cases, while subvalvular and supravalvular account for 20–25% and 5–7%, respectively. AS can occur in isolation or in association with other forms of CHD such as VSD, mitral valve disease, or aortic coarctation (Shone complex).

**Fig. 5:**
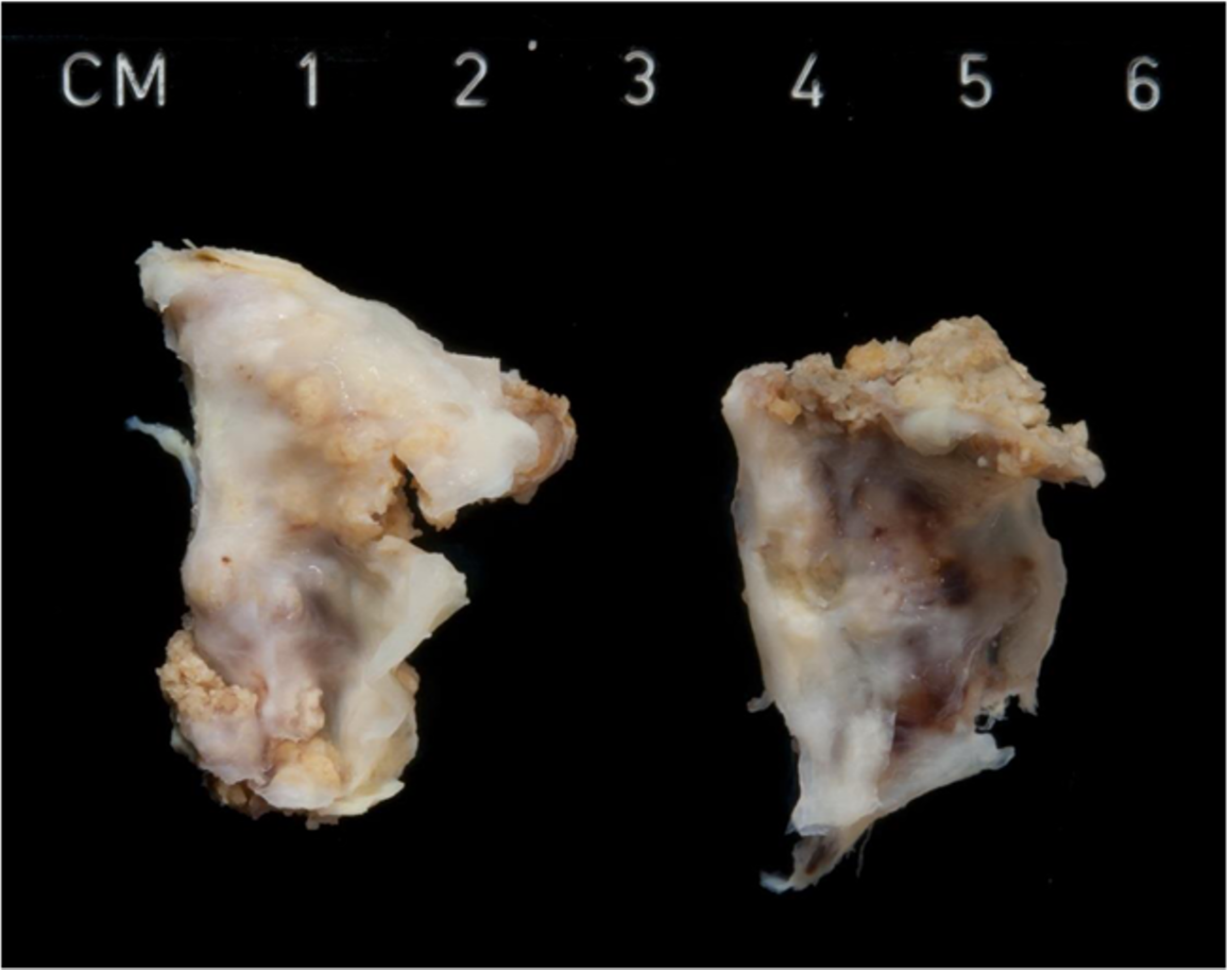
Bicuspid aortic valve in a 50-year-old man, with dystrophic calcification causing aortic valve stenosis

*Valvular stenosis* is the most common cause of bicuspid aortic valve (BAV), with an estimated prevalence of 1–2% in the general population [37]. BAV represents the most common cause of aortic valve replacement or implantation in adult population < 70 years, due to leaflet calcification (Fig. 5). There is complete absence of one of the commissures or presence of a, often heavily calcified, ridge (“raphe”) between two cusps, resulting in a functionally bi-leaflet valve. It is recommended to describe the orientation of commissural anomaly of these valves: absent commissure (or raphe) between the left and the non-coronary (LC-NC) or between the right and the non-coronary leaflets (RC-NC, more frequently associated with aortic coarctation) or between the left and right cusps (LC-RC, more frequently associated with dysfunction and aortic root dilatation). Dilatation of the ascending aorta bears a related risk, albeit still rare, of aortic dissection and rupture with hemopericardium due to medial degeneration. One half of aortic dissections in the youth are associated with BAV.

BAV may be hereditary, and cardiological screening of the family, including echo, is advisable. Much rarer valve abnormalities are the tricuspid aortic valve due to dysplastic leaflets, with thickening or partial commissural fusions or annular hypoplasia; the quadricuspid valve (four normal-looking leaflets or with a double “raphe”); and the unicuspid aortic valve (single cusp with an eccentric “keyhole” or a dome-like appearance) and can be associated with a hypoplastic aortic annulus and ascending aorta. *Subvalvular stenosis* includes a wide range of anomalies from a discrete fibroelastic membrane/ring to a tunnel-like fibromuscular band. It can be complicated by valvular stenosis or with other abnormalities of the subaortic region, such as anomalous accessory mitral leaflet or accessory muscular band. *Supravalvular stenosis* is characterized either by a discrete focal stenosis at the sinotubular junction due to thickening of the aortic wall (seen in Williams Syndrome) [38], or an isolated fibrous membrane, or diffuse tubular hypoplasia of the ascending aorta and brachiocephalic vessels.

Patients may present very early in life or remain asymptomatic for many years. Progression of AS depends on the anatomical features of the valve and the degree of superimposed degenerative calcification or atherosclerosis. Symptomatic lesions in adults are surgically treated, with valves sent to the pathologist as a surgical specimen, but sometimes be found at autopsy, the cause of a severely hypertrophied left ventricle (or dilatation when the valve is severely regurgitant) (Table 1, Tables S2 and S3 supplement).

#### Autopsy recommendation

At autopsy, the pathologist should identify the type and location of LVOT stenosis or obstruction, including accessory fibrous tissue, aortic valve anatomy, and extent of calcifications and signs of endocarditis. The ascending aortic diameter/circumference and aortic wall thickness should be measured to evaluate stenosis, ectasia/aneurism, and atherosclerosis.

In all cases, native valve anatomy should be evaluated. In patients with aortic dissection spontaneous or procedure related, the presence of a BAV should be recorded (see above). In cases of transcatheter aortic valve replacement (TAVR), the valve bearing stent should be removed carefully.

Histological examination of the aortic wall is recommended, with hematoxylin-eosin and elastic van Gieson stains, to establish type and severity of aortopathy (medial degeneration). The aortic arch should be inspected, to rule out isthmic coarctation.

In all cases, left ventricular size and parietal wall thickness should be assessed (hypertrophy, dilatation, scars) also in relation to the RV dimensions. The diameter of the aorta at the aortic annulus (at the level of ventriculo-arterial junction), sinotubular junction, and tubular portion of the ascending aorta should be recorded.

The results of percutaneous valvuloplasty, surgical valvulotomy, and percutaneous or surgical replacement should be evaluated:

1. Acute or short-term procedure-related complications: Hemorrhages (paravalvar), hematomas (check topographic relation with conductive tissues); aortic root lacerations, dissection, thrombosis, and endocarditis; correct position of implant, patency, and (paravalvar) leakage; and thromboembolic complications.
2. Long-term complications (> 6 weeks): Correct position of implant, patency/stenosis, paravalvar leakage (probe), and ingrowth/encapsulation; endocarditis and thromboembolic complications; and excessive fibrosis and calcifications. Always check entire LVOT and mitral valve (specifically anterior leaflet).

A cerebral autopsy is recommended to evaluate potential thromboembolic complications. Eventual associated syndromic pathology should be recorded.

Cardiogenetic consultation should be advised or recommended, both in the proband and relatives, when hereditary disease is suspected (Table 1, Tables S2 and S3 supplement).

### Coarctation of the aorta (CoA)

Description: CoA is a focal or segmental congenital narrowing of the aorta [22].

**Fig. 6:**
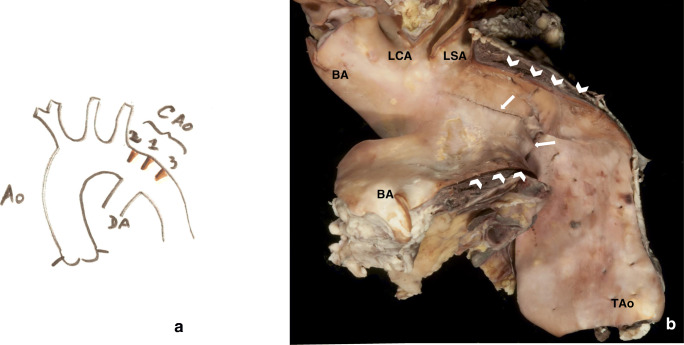
Diagram of the different types of CoA **(a)**. A case of an adult patient with asymptomatic aortic coarctation (arrows without tail), who died because of iatrogenic laceration and dissection (white arrows) of the aorta during percutaneous aortic valve implantation. (TA, thoracic aorta). The patient also had bicuspid aortic valve **(b)**

It usually occurs near the *ductus/ligamentum arteriosum*, in a para-, pre- or postductal location, causing a discrete aortic lumen narrowing or a hypoplastic segment of the aorta [22, 39, 40] (Fig. 6a). Often, discrete CoA and tubular hypoplasia can co-exist. CoA can occur as an isolated form or be associated with other vascular malformations or syndromes [41]. About 15–20% of affected individuals are asymptomatic until adulthood (Fig. 6b). Without repair, adult CoA has a high mortality rate (75% by the fifth decade of life). Therapeutic approach includes balloon dilatation, stent implantation, or surgery (end-to-end anastomosis, prosthetic patch or subclavian flap aortoplasty, or interposition of graft). All the methods may fail leading to re-coarctation and/or have complications [42–45] (Table 1, Tables S2 and S3 supplement).

#### Autopsy recommendations

Evaluate the size of the aortic arch and isthmus, the thickness of the aortic wall at the site of narrowing. Look for signs of hypertension in the upper part of the body (atherosclerosis of the large- and medium-size arteries), LV hypertrophy, congestive heart failure, coronary artery disease, stroke, endarteritis, cerebral berry aneurysms, and aortic dissection. Histopathological features observed in the unoperated CoA include disorganized or fragmented elastic fibers, extracellular mucoid medial accumulation (MEMA) [45], and in adults myofibroblastic proliferation and atherosclerosis.

In case of death early after repair, lacerations of the aortic wall, adventitial hematomas, and thrombosis/thromboembolism should be sought for. Late after repair, false aneurysm and infectious endarteritis (with associated embolic risk), at the site of repair may be encountered (Table 1, Tables S2 and S3 supplement).

### Complete transposition of the great arteries (D-TGA)

**Fig. 7:**
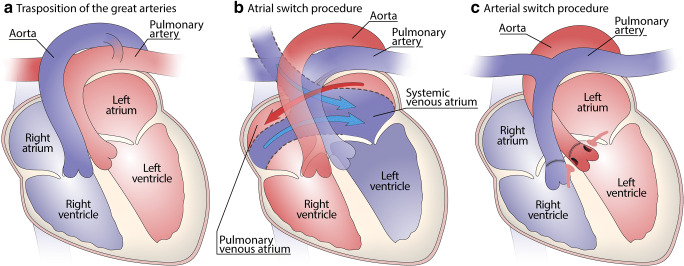
Transposition of the great arteries **(a–c)**: Drawing of the native defect, with the aorta originating from the RV and the pulmonary artery from the left ventricle **(a)**. Drawing of the atrial switch (Mustard or Senning procedure), redirecting the systemic venous blood flow into the left ventricle and the pulmonary trunk, and the oxygenated blood from the lungs into the right ventricle and the aorta **(b)**. Drawing of the arterial switch procedure with repositioning the great vessels above the appropriate ventricles and re-implantation of the coronary arteries **(c)**

Description: Anatomically, the combination of concordant AV connections (the atria are connected to the correct/respective ventricles) and discordant VA connections (the ventricles are connected to the incorrect great arteries) results in systemic venous return being sent to the systemic circulation and pulmonary venous return sent back to the lungs (Fig. 7A).

For survival, a large ventricular or atrial communication is, thus, necessary to allow mixing of blood [46]. Over 60% of cases occur in isolation and require early atrial septostomy; the remaining are associated with other congenital defects, mainly VSD or obstruction of the (subpulmonic) left ventricular outflow tract (LVOT) or both (30%). Most commonly, the aorta is located in right anterior position relative to the pulmonary trunk.

**Fig. 8:**
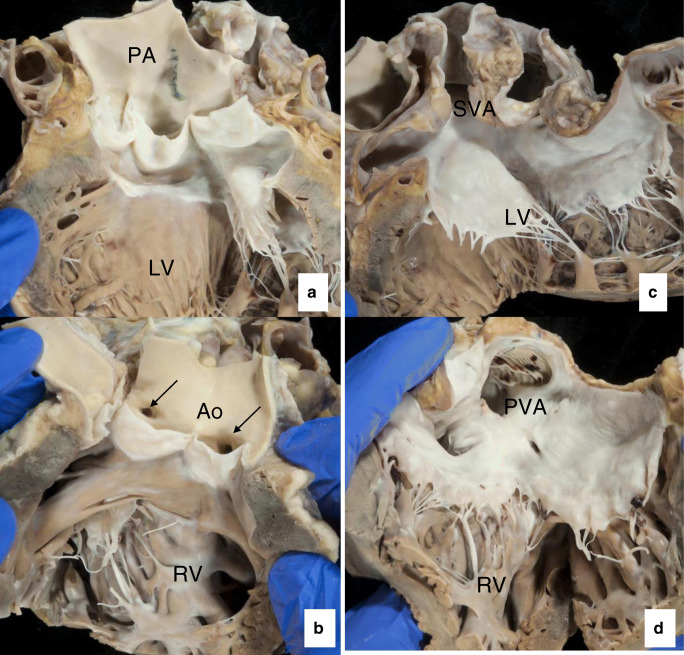
Transposition of the great arteries after Mustard correction at adult age **(a–d)**. Outflow tract of left ventricle is connected to the pulmonary artery **(a)**. Outflow of the right ventricle is connected to the aorta, with “anomalous” origin of the coronary arteries **(b)**. Anterior (atrial) baffle directing the caval blood toward left ventricle **(c)**. Pulmonary venous baffle (with prosthetic material visible) directing blood toward the tricuspid valve and right ventricle **(d)**

In the absence of a large interatrial (ASD) or interventricular communication (VSD), the newborn with complete transposition usually succumbs rapidly upon closure of the oval fossa and the arterial duct due to severe desaturation of the arterial blood. This requires immediate surgical palliation by means of balloon atrial septostomy (Rashkind procedure [47, 48]) to improve hypoxemia. Permanent surgical correction can be achieved at a second stage by means of either *atrial switch procedure* (so-called physiological correction, Mustard [49] or Senning procedure [50], currently abandoned) (Fig. 7B) or, nowadays*, arterial switch procedure* (anatomical correction, Jatene procedure, (Fig. 7C) with excellent early and late-term survival and outcomes) [51] (Fig. 8) (Tables 1 and 2).

**Table 2:** Most important types of systemic to pulmonary artery shunts and definitive repairs

Blalock-Taussig shunt	Subclavian artery directly anastomosed to pulmonary artery (right or left)
Modified Blalock-Taussig shunt	Conduit of synthetic material between subclavian artery and pulmonary artery
Mee procedure	Ascending aorta to main pulmonary artery
Waterston shunt	Ascending aorta to right pulmonary artery
Potts shunt	Descending aorta to left pulmonary artery
Standard ToF repair	Closure of the VSD and relief of the pulmonary/RVOT stenosis
Variants of ToF repair	Right atrial approach versus right ventriculotomy preserving PV versus transannular patch
Rastelli procedure	A Gore-Tex patch tunneling the lef ventricle to the aorta, closing the VSD. The pulmonary valve is surgically closed and an artificial conduit and valve from the right ventricle to the pulmonary bifurcation
Atrial switch procedures (Mustard or Senning)	A two-way baffle in the atria connects the SVC and IVC to the left ventricle and the PV to the right ventricle. In a Senning procedure, the baffle is made with pericardium of the pt.; in the Mustard procedure, a synthetic material is used
Arterial switch (Jatene operation)	The left and right coronary arteries are removed from the Ao and re-anastomosed to the adjacent pulmonary artery, which was to become the neo-aorta, and by transecting and transposing the aorta and main pulmonary artery
Da Silva	Extensive leaflet mobilization, longitudinal plication of the atrialized ventricle, and cone-shaped reconstruction of the tricuspid valve, allowing for leaflet-to-leaflet co-aptation.
Glenn	The SVC is connected to the right pulmonary artery
Fontan	Direct atriopulmonary connection (right atrial appendage to pulmonary trunk which has been detached from the pulmonary valve)
Fontan modified	The IVC is disconnected from the heart and inserted into the pulmonary artery. A conduit can be interposed between the IVC and the PA and can be fenestered. The right ventricle become the systemic ventricle connected the Ao. The SVC is connected to the PA

#### Autopsy recommendations

At autopsy, adult patients are nearly always repaired, and pathology relates to the type of surgical procedure undertaken. *Arterial switch procedure* is presently the preferred choice and performed ideally within 2 weeks from birth. Aorta and pulmonary trunk are transected close to their valves for re-implantation; hence, the pulmonary valve becomes the “neo-aortic” valve, and the aortic valve becomes the neo-pulmonary valve. This procedure also requires (microsurgical) retrieval of the proximal coronary arteries, with re-implantation of their ostia over the neo-aortic valve. In addition, the procedure includes the reconstruction of the pulmonary trunk with pericardial patch (Table 2). Long-term complications include aortic root dilatation with possible aortic valvar insufficiency, left ventricular failure, and myocardial ischemia. RVOT obstruction, and stenosis of branching pulmonary arteries, can occur when the pulmonary trunk had to be repositioned anterior to the aorta (LeCompte procedure). Inspection of coronary ostia and proximal course of the coronary arteries (scarring) is important to rule out kinking and/or acquired atresia.

*The atrial switch procedure* (Mustard or Senning) (Fig. 7B, Table 2) is now largely abandoned in favor of arterial switch (Fig. 7C) and is commonly encountered in older adults with D-TGA. This procedure includes resection of the atrial septum, which is replaced by a “baffle” that channels the superior and inferior caval blood to the pulmonary circulation via the subpulmonary left ventricle, whereas the pulmonary venous blood is channeled from behind the baffle toward the tricuspid valve and systemic RV and tricuspid valve.

On autopsy, severe hypertrophy and dilatation of the RV are observed, with tricuspid insufficiency and deviation of the septum toward the LV unless LVOT obstruction is present. Surgical damage to conduction system, extensive fibrosis due to ventricular remodeling of the RV, and atrial scarring due to surgery are substrates for the frequently occurring atrial and ventricular arrhythmias and instances of sudden death. In addition, the presence of baffle stenosis or leaks must be evaluated, occurring in a quarter of patients. Finally, the presence of endocarditis should be assessed.

*Cases of unrepaired TGA*, typically with a large VSD, are rare in adult life and are typically associated with Eisenmenger syndrome, in the absence of pulmonary stenosis, i.e., severe pulmonary vascular disease (Tables 1 and 2, Tables S1, S2 and S3 supplement).

### Congenitally corrected transposition of great arteries (CCTGA)

Description: Discordant atrioventricular and ventriculo-arterial connections (double discordance) result in a “physiologically correct” circulation through the heart in the absence of associated defects.

However, long-term outcome depends on adaptation of the RV and tricuspid valve to support the systemic circulation, similar to what was described for TGA patients after atrial switch repair [52]. Commonly associated defects include a VSD, pulmonary stenosis, and “Ebstein-like” malformation or other tricuspid valve pathology (15–80%) causing regurgitation [53]. In the absence of associated defects and a well-adapted systemic RV, ccTGA can remain asymptomatic for decades and may be diagnosed very late in life or at autopsy. However, the intrinsic abnormality of the AV conduction system, with the AV node aberrantly located anteriorly and the His bundle on the pulmonary outflow, puts ccTGA patients at risk of wear and tear injury, which explains onset of AV block and cardiac arrest. Reportedly, one half of this patient population requires a pacemaker [54] (Table 1).

#### Autopsy recommendations

Autopsy should start with establishing the viscero-atrial situs, atrioventricular and ventriculo-arterial connections. The position of the aorta in relation to the pulmonary trunk should be recorded, as well as eventual associated lesions. The wall of the morphologic (subpulmonary) LV is typically thin, while the morphologic (systemic) RV is dilated and severely hypertrophied. Tricuspid regurgitation with either abnormal valve leaflets (Ebstein-like) or due to annular dilatation is a frequent complication in the adult, with right ventricular (systemic) failure occurring in patients > 50 years or earlier and an increased risk of endocarditis.

When examination of AV conduction system is required, it should be noted that, in hearts with situs solitus, the coronary sinus and triangle of Koch are not a point of reference for sampling tissue blocks (Table 1).

### Ebstein anomaly of the tricuspid valve

Ebstein anomaly (EA) is rare (< 1% of all CHD cases). It usually occurs sporadically, but familial cases have been reported.

Description: The anatomical hallmark is displacement of the tricuspid hinge line (annulus) toward the RV apex affecting the septal and posterior leaflets.

This apical displacement of the valve results in the pretricuspid part of the RV becoming “atrialized” and thin-walled [55]. Ebstein anomaly is often associated with an atrial septal defect or patent foramen ovale, which may allow left to right shunting and cause systemic desaturation. Wolff-Parkinson-White syndrome (WPW) is present in up to 30% of patients. RV outflow tract obstruction is more often observed in young patients.

Symptoms depend on the severity of tricuspid regurgitation and size of the “functional” RV. This size is variable but can be limited to the outflow tract only, which then becomes dilated and hyperdynamic. Accordingly, the clinical presentation may vary from asymptomatic in very mild cases to deep cyanosis and heart failure (mostly in neonatal cases). Arrhythmias are common in adult life, and there is an increased risk of sudden death [56]. Wolff-Parkinson-White syndrome (WPW) is associated in up to 30% of patients.

Surgical repair with implication of the atrialized portion of the RV and closure of associated septal defects is the preferred option. Currently, the most commonly used technique is the “cone” reconstruction technique described by Da Silva, [57] (Table 2), which creates a cone-looking tricuspid valve from the available leaflet materials [58]. Rarely, when reconstruction is impossible, the valve is replaced with a bioprosthetic valve. Some patients may also undergo bidirectional Glenn anastomosis (anastomosis of the superior vena cava to the right pulmonary artery) to reduce RV preload and the risk of heart failure (Table 1).

#### Autopsy recommendations

Patients presented at autopsy can be either operated at young age (infants, severe disease), operated at older age, or not operated at all. Typically, dilatation of the RV, tricuspid valve annulus, and functional RV is observed. The valve annular dilatation can reach 20 cm and is best visualized via an atrial view, followed by opening of the RV outflow, from the apex to the pulmonary valve. From this incision, the cone reconstruction can be visualized optimally. The displaced leaflets, which have dysplastic appearance, are usually attached to the ventricular wall; the annular attachment of the anterior leaflet is normally located, but the leaflet is often dysplastic and abundant (sail like). Complications include infective endocarditis (operated and non-operated cases), systemic emboli, and pathology related to ablation of arrhythmias (WPW syndrome) or degeneration of a prosthetic valve. Not infrequently, left-sided heart pathology can also be present: ventricular dilatation, fibro elastosis, and left ventricular fibrofatty substitution. Other valvar abnormalities, such as mitral valve prolapse and a bicuspid aortic valve, have been reported (Table 1).

### Anomalous pulmonary venous connections (return)

Description*:* One or more pulmonary veins connect to a site other than the morphologically left atrium.

It can be *total* (diagnosed and repaired early in life) or *partial*, when a solitary vein or all the venous connections from one lung drain into the right-sided atrium or the superior vena cava. Most frequently in adults, the right pulmonary veins drain into the superior vena cava, often associated with the sinus venosus ASD, or the right pulmonary veins drain into the right-sided atrium. In the “scimitar syndrome,” one or two right-sided pulmonary veins “pierce” the diaphragm and drain into the inferior vena cava, and there is dextroposition of the heart because of hypoplasia of the right lung; part of the lung can be sequestrated in terms of its bronchial supply; anomalous pulmonary arterial supply through systemic collateral arteries derived from the descending aorta. In other cases of partial anomalous pulmonary venous connection, the left pulmonary veins drain into the common venous truncus so that the blood enters the right atrium. The presence of an ASD allows the survival of patients with total anomalous pulmonary venous connection, with blood shunting the systemic circulation.

The timing of surgery depends on the severity of obstruction to venous return and oxygen saturations. Partial anomalous connections of the pulmonary veins may be of little functional significance and can be an incidental finding or can become symptomatic later in adulthood. Surgical repair “re-routes” pulmonary venous return to the left atrium using an autologous pericardial patch to create a tunnel in the right atrium through the ASD (Table 1).

#### Autopsy recommendations

In surgically treated adult patients, the pathologist should exclude pulmonary venous obstruction and residual lesions. In cases of scimitar syndrome, the inferior vena cava should be examined, as well as bronchial and pulmonary arterial supply to the right lung. In unrepaired cases, left to right shunting typically results in right heart dilatation. Histology of the lung vasculature is required to exclude pulmonary vascular disease (Table 1).

### Univentricular hearts after a Fontan-type procedure

**Fig. 9:**
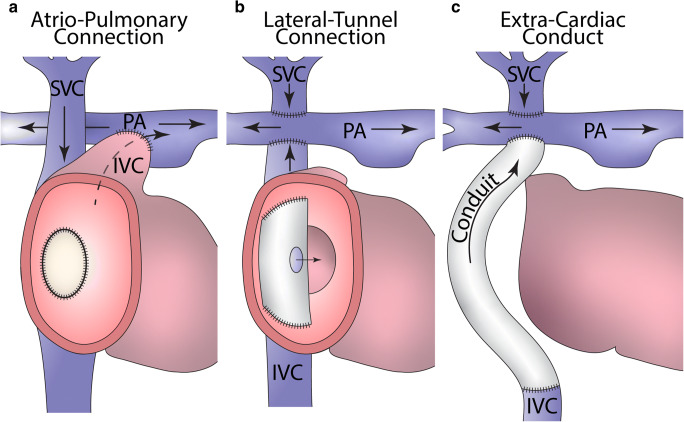
The different types of Fontan-type procedures, grouped in main patterns **(a–c)**. Direct atriopulmonary connection without interposition of conduits **(a)**. Intracardiac lateral tunnel draining the IVC into the pulmonary circulation bypassing the right ventricle **(b)**. Extracardiac conduit draining the IVC blood into the pulmonary arteries **(c)**. In **(b)** and **(c)**, a bidirectional Glenn anastomosis drains SVC blood to the right pulmonary artery

Named after Francis Fontan, a brilliant French surgeon who invented the procedure in 1968, the Fontan procedure and the many modifications over the years are palliative surgical interventions designed for patients who cannot undergo biventricular repair [59] (Table 2). There are many types of congenital anomalies that can be palliated by the Fontan and modified Fontan procedures all of which have in common the feature of a *univentricular physiology*, whereby only one ventricle can be used to support the systemic circulation (Fig. 9). For example, in hypoplastic left heart syndrome, the LV is too small or inadequate to support the systemic circulation so the RV is, therefore, recruited to support the systemic circulation instead. Other such heart malformations are tricuspid atresia, pulmonary atresia with intact ventricular septum, hypoplastic left heart syndrome, double-inlet left or right ventricles, atrioventricular septal defect with unbalanced ventricles, and single ventricle with undefined morphology. The outcomes after these palliative procedures are nowadays optimal when performed at a young age, with an operative mortality of 5% and a midterm mortality at 10 years of approximately 10% depending on the type of defect. However, long-term complications are common and relate to the lack of a subpulmonary ventricle, which produces chronic systemic venous hypertension and a preload deprived systemic ventricle. The modes of death vary: perioperative (68%), sudden cardiac death (9%), thromboembolism (8%), heart failure (7%), and sepsis (3%) [60]. Heart failure is becoming the most common cause of death in adult patients and those with a systemic RV (Table 3). Liver complications are common late after Fontan operation, including cirrhosis and hepatocellular carcinoma. Protein-losing enteropathy and plastic bronchitis are also complications of the Fontan circulation.

The aim of a Fontan-type operation is to route the systemic venous blood flow directly into the pulmonary artery without the interposition of a ventricle. This can be achieved in different ways. The oldest procedure implies a direct atriopulmonary connection (right atrial appendage to pulmonary trunk which has been detached from the pulmonary valve). More recently, the Fontan procedure is performed by means of a total cavopulmonary connection, with an intracardiac tunnel or an extracardiac conduit, draining the blood flow from the inferior vena cava to the pulmonary artery, and with a bidirectional cavopulmonary shunt (termed bidirectional Glenn shunt), draining the blood flow from the superior vena cava directly to the confluent pulmonary arteries. The Fontan pathway may be fenestrated, allowing communication with the left-sided atrial chamber, as an escape (relief valve) mechanism in case of increasing pressures following intervention (Fig. 9).

#### Autopsy recommendations

**Fig. 10:**
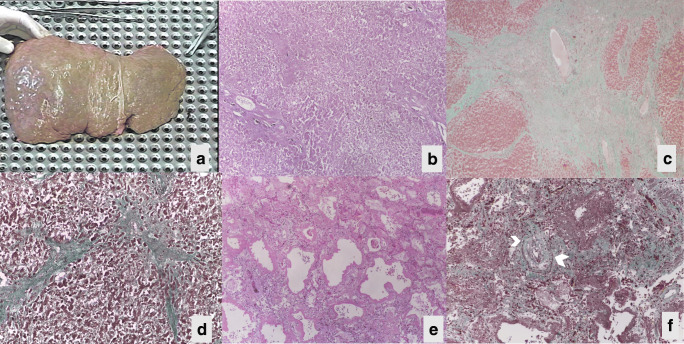
Failing Fontan circulation with hepatic cirrhosis and pulmonary hemorrhage **(a–f)**: macroscopic view of the liver at autopsy, with evidence of cirrhosis **(a)**; histology showing fibrosis with disorganization of the structure, dilatation of the veins, and regenerative nodules using hematoxylin-eosin staining **(b)**; Masson trichrome staining highlighting the fibrosis in green **(c)**; high-power view of c **(d)**; lung hemorrhage, hematoxylin and eosin staining **(e)**; lung thrombosis (white arrows), Masson trichrome staining **(f)**

Late complications after Fontan procedures that should be evaluated at autopsy include Fontan pathway obstruction (anatomical or thrombosis), right atrial dilatation, pulmonary artery stenosis or thrombosis, pulmonary vascular disease, atrioventricular valve regurgitation, and pulmonary venous obstruction. A “failing Fontan” circulation is also characterized by congestive heart failure with peripheral congestion, ascites, pleural effusions, and multi-organ involvement, including hepatic changes (hepatomegaly, cirrhosis, portal hypertension, and hepatocellular carcinoma), protein loosing enteropathy, and renal failure (Fig. 10a–c)**.** Chylothorax and plastic bronchitis can also occur in the medium term. Pulmonary thrombosis and hemorrhage (Fig. 10d), arteriovenous malformations, and venovenous collaterals are not uncommon. A restrictive VSD may act as LVOT obstruction in cases with transposed great arteries (Tables 1 and 2, Tables S1–S3 supplement).

### Coronary artery malformations

Description: Congenital malformations of the coronary arteries are characterized by aberrant origin, course, or size of epicardial arteries.

They occur either as isolated anomalies or in combination with more complex congenital heart disease. Some of these are relatively benign lesions, but others have an increased risk of sudden death already early in life. For a detailed description of the anomalies in this chapter, see the manuscript published by the Development, Anatomy, and Pathology of the European Society of Cardiology (ESC) working group [61]. An anomalous origin of the left coronary artery from the pulmonary artery (ALCAPA) is a rare high-risk condition that presents early in life and causes early death in infancy unless repaired. However, rare cases of SCD have been reported even in adulthood [62].

**Fig. 11:**
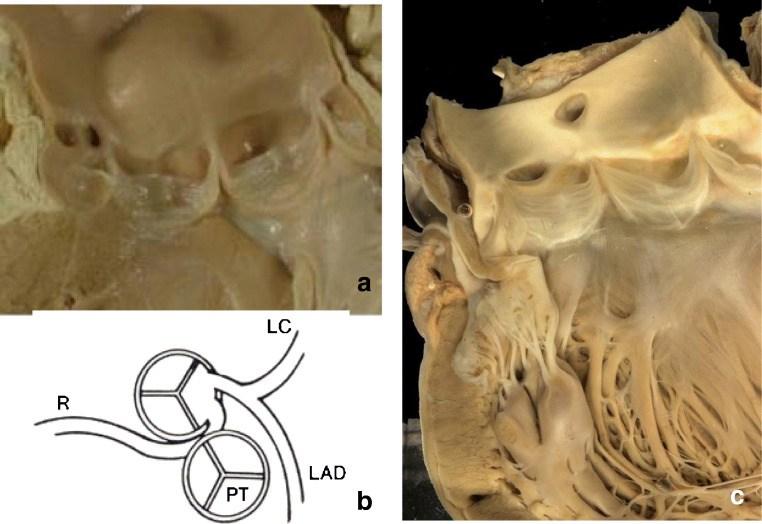
Congenital coronary arteries anomalies in two adult patients. Left coronary artery originating from the wrong sinus, running in front of the pulmonary trunk, at low risk of SCD **(a)**; drawing of a coronary artery anomaly with abnormal course between the aorta and the pulmonary arteries, at high risk of SCD **(b)**. High takeoff of the right coronary artery above the sinotubular junction, at low risk of SCD **(c)**

Origin of the left or right coronary artery from the opposite (wrong) sinus is considered a low risk condition (Fig. 11a), except when there is anomalous (interarterial) course between aorta and pulmonary artery, in which case it is regarded as a “high-risk” anomaly associated with SCD (Fig. 11b). The so-called minor anomalies in coronary development have incidentally also been reported to precipitate arrhythmic cardiac arrest due to myocardial ischemia, mostly during efforts or sports activity.

“High takeoff” of a coronary artery is considered as a low-risk lesion (Fig. 11c), unless the artery exhibits also an intramural (aortic) course, which may cause ischemia during exercise. Coronary ostia originating up to 1.2 cm above the sinotubular junction are considered variants without clinical significance [63].

#### Autopsy recommendations

The site of the coronary ostia and course should be documented: exclude anomalous origin from the pulmonary artery or from a wrong aortic sinus, assess the height and location of the ostia in the ascending aorta, and whether there is an anomalous intramural course or course between the aorta and pulmonary artery. Take a vertical sample at a stenotic site for histological examination. Myocardial histology should evaluate acute (contraction bands) or late patchy fibrosis, indicating ischemic injury in the myocardium at risk (Table 3).

**Table 3:** Autopsy procedures

Appropriate photographic documentation of the hearts is recommended	
*Handling of the heart*: Remove en bloc the heart and lungs with the thoracic aorta for a proper complete evaluation. If a second opinion is sought the entire block, and not only the heart, should be referred (after washout)	Fresh heart Wash out to remove blood clots Put crumpled paper towels or gauze soaked in formalin in ventricle cavities to allow fixation without distortion Take small ventricle pieces to be frozen Put the heart in an appropriately sized container with sufficient 10% formalin to cover For proper valuation of surgical conduits and anastomosis, it is important to investigate the heart lung specimen, with large arteries (pulmonary arteries and aorta including aortic arch intact). When dissecting the heart, it is also important to keep the roof of the atria with inflow of the pulmonary and systemic veins intact
*Gross examination before sectioning the heart*	Describe the heart: dimensions (hypertrophic, dilated, normal) Consider a plain X-ray for localization of devices, catheters, calcifications, etc. *Weight* the heart Measure *longitudinal distance* (distance from the crux cordis to the apex on the posterior aspect) *Transverse distance* (from the obtuse to the acute margin along the posterior atrioventricular sulcus) Possible aneurysms Describe amount and distribution of epicardial fat Examine the atrial cuffs and atrial appendages for size, endocardial lesions and thrombi Examine atrioventricular valves (atrial view) and the semilunar valves (arterial view) and note any abnormalities (mention bicuspid valve)
Non-operated congenital heart disease	
*Sectioning*	Before dissection, check whether the heart is biventricular or univentricular: with univentricular physiology, it is essential to identify the rudimentary ventricle Dissect the heart following blood flow (a transverse cut at mid-ventricle is not usually recommended)
*Gross examination*	Use the *sequential segmental approach* whose main steps are the following: Definition of atrial situs Morphologic identification of atria, ventricles and great arteries Recognition of atrioventricular and ventriculo-arterial connections
*Sampling for histology*	Sample left and right ventricle.
Operated congenital heart disease	
*General recommendations*	Assess any devices, including valves and patches or biological grafts relating to previous surgical or interventional procedures. (endocarditis, thrombosis) Check for catheters within the cavities and epicardial leads
*Sectioning*	Structure of the heart can be markedly altered; it is advisable to have a clear idea of the primary disease and subsequent surgery before sectioning, if possible with the surgeon or cardiologist present.
*Coronary arteries*	Locate the coronary ostia and/or eventual aberrant course Make transverse cuts at 3-mm intervals in the main epicardial branches to identify atherosclerotic stenosis / occlusion (as usual)
*Sampling for histology*	A complete section of the heart at mid-ventricular level is not recommended In non-operated and operated hearts histological evaluation could be important for dating the injuries or thrombosis or any complications and for assessing the pathological substrates (fibrosis, ischemic injury, endocardial elastosis, scars), which may contribute to cardiac failure and/ or arrhythmias. I would recommend sampling of the heart for interstitial fibrosis and endocardial elastosis of the right and left ventricle. One sample from the right ventricle, one from the left ventricle.
*Staining*	When warranted by hematoxylin-eosin, stains for collagen and elastic fibers (Masson or Mallory trichrome, Movat pentachrome, Wiegert van Gieson, etc.) and additional stains or immunohistochemistry on indication

### Congenital anomalies of the conduction tissue

Congenital AV block and WPW syndrome may be associated with SCD. Usually, diagnosis is accomplished at ECG following syncope, epileptic seizures, or palpitations.

Congenital heart block in adolescents and adults is either related to the underlying condition or the result of surgery or intervention. It may also be precipitated by medication or endocarditis. Indeed, permanent pacing is commonly required in conditions such as those with congenitally corrected TGA or atrioventricular septal defects.

Wolff-Parkinson-White syndrome consists of ventricular preexcitation due to an anomalous AV connection of working myocardium leading to re-entry supraventricular tachycardia. Complicating factors such as atrial myocarditis and very short refractory period of the accessory pathway may lead to ventricular fibrillation and cardiac arrest [64].

#### Autopsy recommendations

Electrocardiograms should be examined from clinical records prior to autopsy. In cases of suspected or established WPW, the tricuspid valve should be examined to rule out Ebstein anomaly, and myocarditis should be excluded. Histologic identification and examination of the accessory pathways are usually not required, because in nearly all cases diagnostic electrocardiogram is available. Systematic histological investigation of such aberrant conduction pathways is very laborious and time-consuming and is not routinely recommended.

## Special considerations in cases of redo surgical procedures

Prior to autopsy, the indications for the intervention and detailed surgical reports should be examined, together with the past medical history. Surgical re-interventions require longer cardiopulmonary bypass and surgery time, impacting on perioperative hemostasis, myocardial, lung, and renal function. Common reoperations are aortic or pulmonary valve replacement, AVSD repair, RV-to-pulmonary artery conduit replacements, Fontan (TCPC) conversion, aortic coarctation, and repairs of Mustard pathways leaks or obstruction (Table 2).

Perioperative mortality in these patients increases with age, the complexity of the anomaly, the number of previous operations, and, not at least, the co-morbid pathology such as renal failure, pulmonary hypertension, and obesity.

### Autopsy recommendations

Previous interventions can result in dense mediastinal adhesions, pericardial adhesions, lung scarring or atelectasis, and chest deformities. Chronic cyanosis can affect hemostasis and promotes perioperative bleeding, but paradoxically also thrombosis, and may also cause renal failure. Causes or contributors to death around redo procedures include ventricular or organ dysfunction,, infections, embolic events (e.g., thrombotic or air emboli), refractory congestive heart failure (e.g., persistent pleural effusion or ascites), migration of devices, laceration or perforation of vital cardiac structures, and injury to adjacent organs (e.g., the lung).

## Pathology of the major clinical manifestations of ACHD

### Heart failure

Heart failure (HF) is very often the clinical evolution in ACHD patients, even those operated early in life in the best clinical setting. HF can be part of the natural history of a defect, and/or a sequela of previous palliation or repair [65]. Heart transplantation is the ultimate option for patients with advanced heart failure refractory to treatment, with particular cases receiving mechanical circulatory support as destination therapy or as a bridge to transplantation [66, 67]. Over 30% of ACHD patients are likely to die of heart failure [68, 69]. All CHD can evolve to HF, but at “high risk” are the patients unrepaired cyanotic lesions and with univentricular hearts (failing Fontan circulation). The most common mechanism for the development of HF is ventricular dysfunction due to severe pressure or volume overload, myocardial ischemia, and prior surgery [70]. Ventricular dysfunction can also be worsened by arrhythmias, which are the result of abnormalities in the conduction system or parietal wall scarring due to surgery [71]. With the increase in survival of ACHD patients, comorbidity also contributes to the development of heart failure, which can be systemic hypertension, coronary atherosclerosis, myocarditis, diabetes mellitus, smoking, or drug abuse. The occurrence of acute heart failure must be also considered. This can be the consequence of rapid progression of preexistent chronic heart failure but can also be due to ischemia, acute coronary syndromes, tachy- or bradyarrhythmias, valvular insufficiency, cardiac tamponade, or pulmonary embolism [7].

#### Autopsy recommendations

Systemic signs of heart failure, such as peripheral edema, pleural effusions, lung congestion, ascites, liver congestion, gastritis, and evidence of kidney failure, should be assessed. Any native CHD, or the type of surgical or interventional procedures, residual lesions, and cardiac remodeling should be described, and the likely mechanisms of heart failure should be identified. Conditions that may precipitate chronic heart failure or determine acute heart failure should also be identified (see above) (Table 3).

### Pulmonary arterial hypertension

Patients with congenital heart disease (CHD) are frequently affected by pulmonary arterial hypertension (PAH), especially when large systemic-to-pulmonary shunts are left untreated or are repaired late, before the onset of irreversible lung lesions [72, 73]. When pulmonary vascular disease becomes severe [74], the shunt reverses (from left to right to bidirectional) and cyanosis appears in the landmarks of Eisenmenger syndrome (ES) [75]. The exact prevalence of PAH in ACHD is unknown, but a European registry reported an estimated prevalence between 5 to 10%, and about 25–50% of these patients had ES [76]. International pulmonary hypertension guidelines classify PAH related to CHD (PAH-CHD) into four categories: ES; PAH associated with prevalent systemic-to-pulmonary shunts; PAH with small/coincidental defects; and PAH persisting, recurring, or developing after defect correction [77].

In general, the parameters associated with the onset and also severity of PAH-CHD are the type and dimension of the congenital defect, direction of the shunt, associated extracardiac anomalies, and the type and timing of surgery (palliative or definitive).

Morbidity and mortality rates are significant in PAH-CHD and are often the result of chronic hypoxia, cardiac dysfunction, and refractory congestive HF or sepsis/infections. In many patients, death may be sudden, due to hemoptysis, pulmonary hypertensive crisis, or arrhythmias of which the latter relate to long-standing cardiac overload or myocardial scars [78]. Acute deterioration of HF can also occur soon after inappropriate late closure of a septal defect or other communication, in ES patients, in whom such defects act as “relief valves” for the RV. Pulmonary causes of sudden onset death in PAH patients are thromboembolic events or hemoptysis due to rupture of small or large pulmonary vessels or bronchial arteries.

#### Autopsy recommendations

The autopsy should start with evaluation of the type of CHD and associated anomalies and complications, such as endocarditis or other infection. Extracardiac causes of death should also be considered, e.g., paradoxical emboli to the brain or brain abscess. Dimensions of cardiac chambers are important to record: degree of RA and RV dilatation and wall thickness of RV for hypertrophy, comparing this to the LV. Potential precipitating factors, such as lung infection, thrombosis, myocardial ischemia and postoperative reperfusion injury should be evaluated. Macroscopic evidence of long-standing PAH includes presence of dilated pulmonary arteries, with significant atherosclerotic plaques (which can be even aneurysmatic and ulcerating in severe cases) at the level of pulmonary trunk and pulmonary arteries and stratified (in situ) thrombus.

**Fig. 12:**
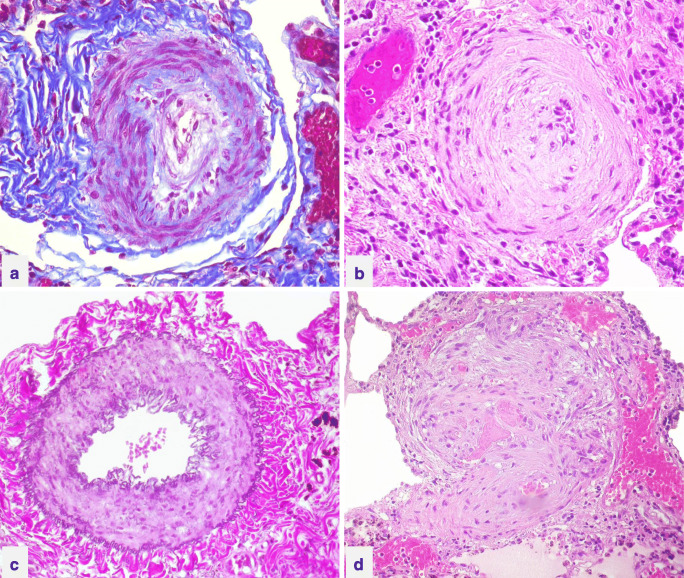
Histology of vascular remodeling in pulmonary arterial hypertension **(a–d)**. Concentric non-laminar intimal thickening Azan Mallory staining **(a)**, hematoxylin-eosin staining **(b)**; medial muscular hypertrophy, Wiegert van Gieson staining **(c)**; plexiform lesions, hematoxylin-eosin staining **(d)**

Histology of each lobe is required to assess pulmonary vascular disease. For correct handling of tissue, the lungs should be fixed in a state of distension to avoid artifacts in elastic laminae and exaggerated medial hypertrophy. The main histological changes, in increasing order of severity, are concentric intimal thickening, muscular hypertrophy and dilatation of arterioles and small arteries, and the formation of “plexiform lesions” in keeping with Heath-Edwards classification. Fibrinoid necrosis is considered as an acute lesion, secondary to intense vasoconstriction and endothelial damage with insudation of fibrinogen into the arterial wall. Hematoxylin-eosin and elastic van Gieson stains are essential; immunohistochemical staining for smooth muscle cells (actin, desmin) and endothelial cells (CD31, CD34) can sometimes be of help to delineate the vascular pathology. The current pathological classification of vasculopathies in PH was proposed in 2003 at the Third World Symposium on Pulmonary Hypertension [79] (Fig. 12) (Table 3).

### Sudden cardiac death

Sudden cardiac death (SCD) is not uncommon in both repaired and unrepaired/palliated ACHD patients (see also AECVP guidelines on SCD) [80].

The mechanism of cardiac arrest is in most cases arrhythmic, in the setting of severe electrical instability of the heart with a complete heart block or life-threatening ventricular arrhythmias arising in the “working” myocardium. Cases of “mechanical SCD” are rare and include hemorrhages due to aortic or pulmonary artery ruptures or dissections and (thrombo) embolization or sudden death in patients with severe obstructive lesions [81].

For practical purposes, there are three categories of patients in whom SCD may occur:

1. *Overt CHDs since birth or unrepaired CHD.* This group includes patients with unrepaired univentricular hearts or with large septal defects who have developed significant pulmonary vascular disease (ES).
2. *Overt CHD since birth, repaired surgically or percutaneously.* Patients with repaired tetralogy of Fallot carry a small but well-documented risk of sudden death, often related to the scarring of the RV outflow tract as a consequence of infundibulotomy with transannular patch or RV pulmonary artery conduit implantation. Patients with systemic ventricular dysfunction (e.g., patients with a systemic RV after atrial switch for TGA) and those with Ebstein anomaly are at risk of sudden arrhythmic death. Surgical or percutaneous repair of a VSD and endocarditis of the aortic valve can affect the conduction system, causing complete heart block.
3. *Congenital heart disease diagnosed in adult life*. There are congenital cardiac anomalies that are not symptomatic at birth, infancy or childhood. They remain silent and may be diagnosed by chance, when an ECG or echocardiogram is performed later for screening or other reasons. There are four main categories to be excluded on autopsy: coronary artery anomalies, conduction tissue defects, CCTGA, and the bicuspid aortic valve (as described above).

#### Autopsy recommendations

See AECVP guidelines on SCD [80].

## Conclusion

Significant knowledge and expertise are required to perform an autopsy in an ACHD patient. This document provides an overview of the most common types of ACHD and potential autopsy findings including its interpretation in relation to the clinical scenario at the time of death. As such, it may serve to guide postmortem examinations performed by non-CHD pathologists who need to perform autopsy in ACHD patients (Table 3, Box 1–2). Close collaboration with tertiary CHD centers is strongly recommended, discussing complex cases and ensuring high-quality outcomes for clinical, medicolegal, and educational purposes.

Box 1

**Table:** 

**Template for Autopsy Report in Adult Congenital Heart Diseases**	
The pathology report should include a descriptive section and the final diagnosis.	
***Descriptive section*** should include:	
• A sufficiently detailed clinical history with the native congenital heart disease, time and type of surgical and hybrid procedures.	
• Systematic description of the macroscopic findings at autopsy.	
• Systematic description of the microscopic findings, if required.	
• Description of any additional tests, if performed.	
To understand the gross autoptic findings, the pathologist at autopsy should be aware of the cardiac morphology in the different types of CHD, the pathophysiology and the possible surgical or interventional techniques for palliation or repair. Microscopic and molecular findings should correlate to the macroscopic autopsy findings before interpretation can be finalized. Before the release of the report the pathologist should always discuss findings with the surgeon and the cardiologist.	
***Final diagnosis*** should include:	
• Cause(s) of death.	
• Description of the native congenital heart defect and status after surgical correction. (state remodeling of the heart, which includes also histological appearance, chambers after surgery, and other complications)	
• Pathologic findings in order organs (in order of priority)	
• Description of the surgical and interventional procedures	
• Epicrisis or clinicopathological correlations and comments.

**Table:** Box 2

**Questions to be answered after autopsy:**	
What was the cause of death?	
Was the preoperative diagnosis correct?	
Could the death have been avoided?	
Appropriate indication to repair or palliation? Timing of surgical or interventional procedures?	
Evaluation of surgical or interventional techniques.	
Appropriate perioperative management.	

## Electronic supplementary material

ESM 1 (DOCX 15 kb)

## Abbreviations

ACHD: adult congenital heart disease
CHD: congenital heart diseases
SCD: sudden cardiac death
ASDs: atrial septal defects
FO: fossa ovalis
SCV: superior caval vein
ICV: inferior caval vein
CS: coronary sinus
LA: left atrium
RA: right atrium
RV: right ventricle
LV: left ventricle
AVSDs: atrioventricular septal defects
VSDs: ventricular septal defects
CAT: common arterial trunk
RVH: right ventricular hypertrophy
ToF: tetralogy of Fallot
PA: pulmonary artery
PV: pulmonary valve
ICD: implantable cardioverter defibrillator
AS: aortic stenosis
LVOTO: left ventricular outflow tract obstruction
BAV: bicuspid aortic valve
LC-NC: left coronary-non-coronary
RC-NC: right coronary-non-coronary
TAVR: transcatheter aortic valve replacement
CoA: coarctation of the aorta
D-TGA: complete transposition of the great arteries
AV: atrioventricular
VA: ventriculo-arterial
RVOT: right ventricular outflow tract
CCTGA: congenitally corrected transposition of great arteries
EA: Ebstein anomaly
WPW: Wolff-Parkinson-White
ESC: European Society of Cardiology
ALCAPA: anomalous left coronary artery from pulmonary artery
TCPC: total cavo pulmonary connection
HF: heart failure
PAH: pulmonary arterial hypertension
AECVP: Association for European Cardiovascular Pathology
TV: tricuspid valve
