# Supplementary material for: Autopsy in adults with congenital heart disease (ACHD)
Source: Virchows Arch. 2020 Apr 7;476(6):797–820. doi: 10.1007/s00428-020-02779-8 (PMC7272495; doi:10.1007/s00428-020-02779-8)
Supplement: Supplementary file 1 — (DOCX 15 kb) [file 428_2020_2779_MOESM1_ESM.docx]

**Table S1: Common late complications after Fontan-type procedures (supplement)**

Failing Fontan circulation: Low cardiac output state with ascites, pleural effusions, peripheral edema, plastic bronchitis, severe cyanosis. Potential causes:

- Narrowing or distortion of the TCPC/Fontan pathways (intra or extracardiac).

- Pulmonary artery hypoplasia, stenosis or thrombosis

- Pulmonary vein stenosis

- Systemic ventricular dysfunction (systolic or diastolic)

- Systemic atrioventricular or aortic valve regurgitation, outflow tract obstruction (restrictive VSD with transposed great arteries)

- Right atriomegaly (atriopulmonary Fontan), right atrial thrombus

- Recanalization of the ligated pulmonary trunk (competing flow with the Fontan circulation)

*Additional findings:*

- Hepatomegaly or other hepatic changes (fibrosis, cardiac cirrhosis, hepatocellular carcinoma)

- Systemic venous collaterals (acquired or preexisting, i.e. prior to surgery)

- Protein losing enteropathy (4-13% of the patients).

- Complications of interventions (fenestration closure, occlusion of collaterals, relief of pulmonary stenosis), temporary pacing (transaortic approach)

- Endocarditis

**Table S2 : Interventional procedures in ACHD and potential complications (supplement)**

| **Type of procedures** | **Devices** | **Complications** |
| --- | --- | --- |
| Closure of patent ductus arteriosus | PDA occluder or coils | Small residual defects, Thrombosis of the device, systemic emboli, wire or catheter related complications |
| Closure ASD | Occluders in the atrial septum | Embolization of the device, device migration or erosion, pulmonary vein obstruction, atrial septal hematoma, mitral or aortic valve regurgitation, infective endocarditis, cardiac perforation, stroke, lung congestions |
| Closure VSD | Occluders in the ventricular septum | Residual defect, interference with the aortic or AV valves, thrombosis, arrhythmia, wire or catheter related complications |
| Closure of coronary fistulas, pulmonary vascular malformations and aorto-pulmonary collaterals, and veno-venous collaterals | Occluders/coils in the vessels involved | Residual defect, perforation of the vessels involved |
| Closure of paravalvular leaks | Occluder close to the prosthetic valve previously surgically or percutaneously implanted | Interference with valve function, thrombosis, embolization, arrhythmia, wire or catheter related complications |
| Angioplasty & stenting of pulmonary artery, | Stent in the main pulmonary and distal/peripheral arteries | Intrastent thrombosis, stent dislodgement or inaccurate positioning, hematoma of the parietal wall, rupture of the treated vessels |
| Angioplasty & stenting for coarctation of the aorta | Stent in the aortic isthmus |  |
| Angioplasty & stenting of pulmonary veins | Stent in the pulmonary veins |  |
| Angioplasty & stenting of surgical conduits, and baffles | Stent in surgical conduits (e.g. RV-to-pulmonary artery, total cavo-pulmonary connection) |  |
| Angioplasty & stenting of the interatrial septum and Fontan fenestrations | Stent in atrial septum and Fontan conduit |  |
| Transcatheter pulmonary valve and valve-in-valve or valve-in-conduit implantation | Transcatheter valve in the pulmonary position | Valve or stent embolization (particularly when advancing the valve through a pre-existing stent), conduit rupture with hemodynamic compromise, PA perforation, coronary artery compression, and obstruction of either PA by the stent or device |
| Transcatheter tricuspid valve-in-valve implantation | Transcatheter valve in tricuspid valve position | Malposition of the prosthetic valve |
| Transcatheter aortic valve implantation (in aortic stenosis or bioprosthetic valve) | Transcatheter valve in aortic valve position | Residual outflow tract obstruction. Atrioventricular node and conduction tissue damage, laceration of the aorta  Long term valve cusps calcification /degeneration |

**Table S3 : Hybrid ACHD procedures (supplement)**

| **Type of abnormalities** | **Devices and procedures** |
| --- | --- |
| **PDA closure** | Endovascular Stent graft at the level of PDA+ Surgical closure at the pulmonary site |
| **Coarctation of the aorta** stenting | Covered stent at the level of the CoA+ Surgical re-implantations of head and neck vessels |
| **Pulmonary venous** baffle obstruction or leakage after atrial switch. | Stent insertions for stenosis or occlude in case of baffle leakage |
| **Fontan fenestration closure**  **Or Fontan baffle puncture for fenestration** | Septal occlude or covered stent implantation |
| **Tricuspid valve implantation for TV Bioprosthetic valve dysfunction**  **Percutaneous Pulmonary valve implantation in RV outflow tract obstruction or dilatation** | Transcatheter valve implantation in TV  Pulmonary Transcatheter valve implantation in dysfunctional synthetic RV-PA conduit, pulmonary Homograft, Bioprosthetic valve  Surgery for reduction of the outflow tract+ Percutaneous Transcatheter valve implantation |
| **Repair of paravalvular leak** | Occluder in relation to the leakage+ previous bioprostetic surgical replacement |
